# Supplementary material for: The distribution of beneficial mutational effects between two sister yeast species poorly explains natural outcomes of vineyard adaptation
Source: Genetics. 2024 Oct 7;228(4):iyae160. doi: 10.1093/genetics/iyae160 (PMC11631397; doi:10.1093/genetics/iyae160)
Supplement: iyae160_Supplementary_Data [file iyae160_supplementary_data.zip › Supplemental_Figures_GENETICS-2024-307376.docx]

Supplementary figures for

“The distribution of beneficial mutational effects between two sister yeast species poorly explains natural outcomes of vineyard adaptation”

Emery R. Longan and Justin C. Fay


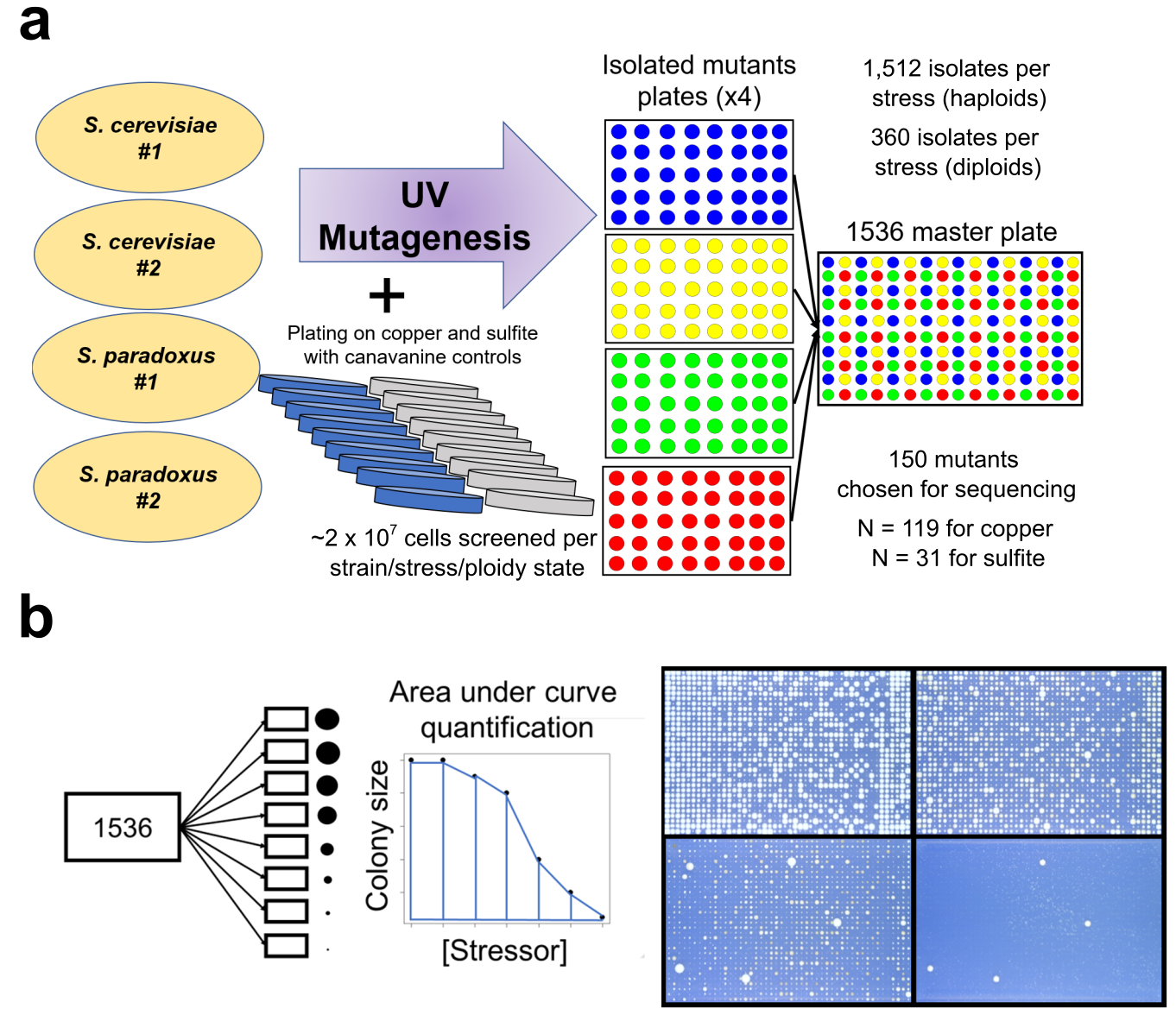


**Figure S1**. Schematic of the mutant isolation and phenotyping process. (a) For haploids and diploids, four ancestral strains were used that were sensitive to copper and sulfite. These strains were then UV mutagenized, and the mutagenized pools were plated onto plates with various concentrations of copper and sulfite. Canavanine was used as a control for mutation rate in haploids. Next, mutants derived from each ancestor were manually arrayed in a grid. We used 384 format grids for haploids and 96 format grids for diploids. Then these isolated mutants were collapsed to a master plate to await phenotyping. Following phenotyping, 150 mutants were selected for genome sequencing. (b) Schematic of our AUC phenotyping method. The 1536 master plate is replica plated onto many concentrations of stressor using a Singer Rotor HDA robot (Singer Instruments). After growth, the size of each colony on each plate is quantified via imaging each plate and measuring the number of pixels occupied by each colony. These colony size measurements for each position on the plate are collapsed to a single data point by measuring the area under the curve of colony size as a function of stressor concentration using the trapezoid rule. For diploids, sulfite mutants and copper mutants were phenotyped together with two technical replicates.


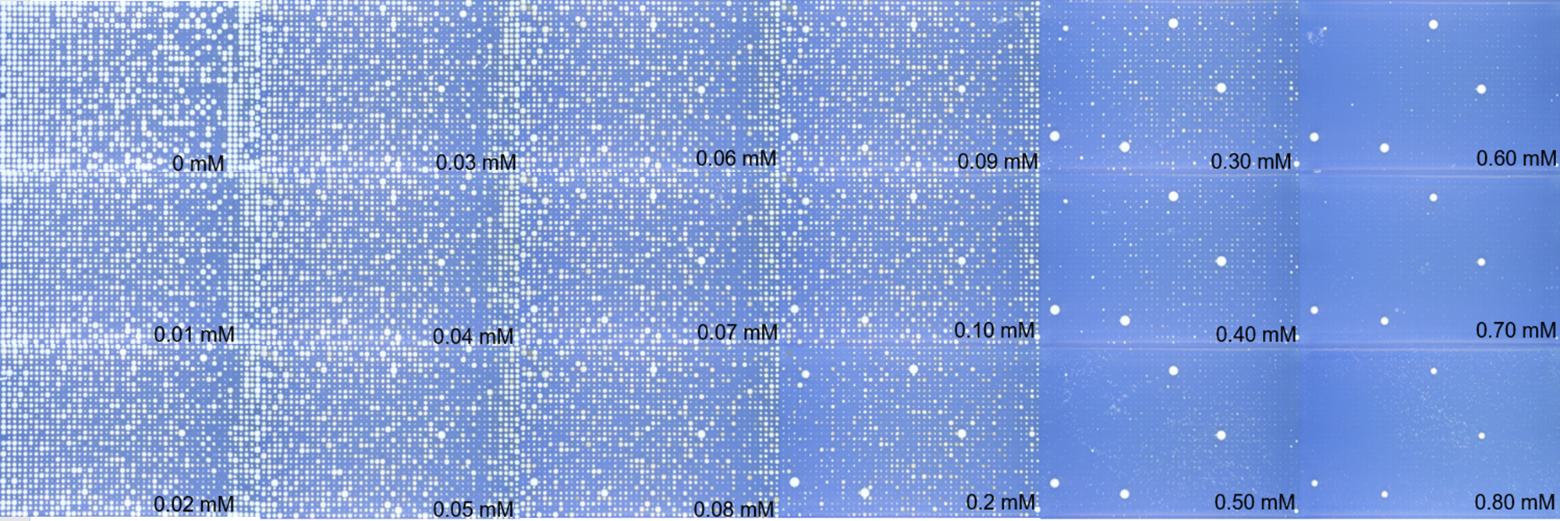

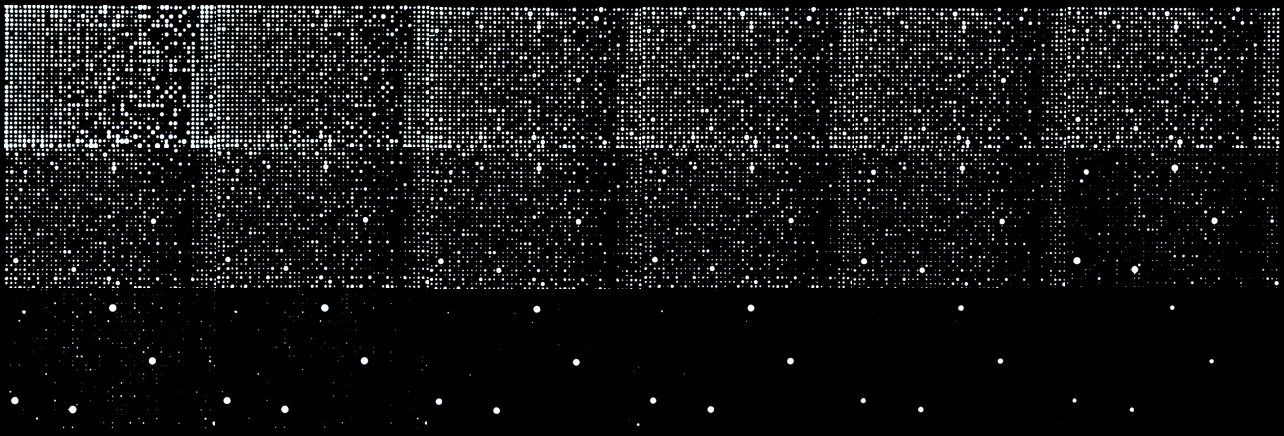


**Figure S2**. Example of colony size quantification method from the haploid copper mutants. Each image is a 1536 format plate with varying amounts of copper sulfate added (top). When the plates are imaged, we used background subtraction and pixel quantification to measure the area under the curve (bottom).


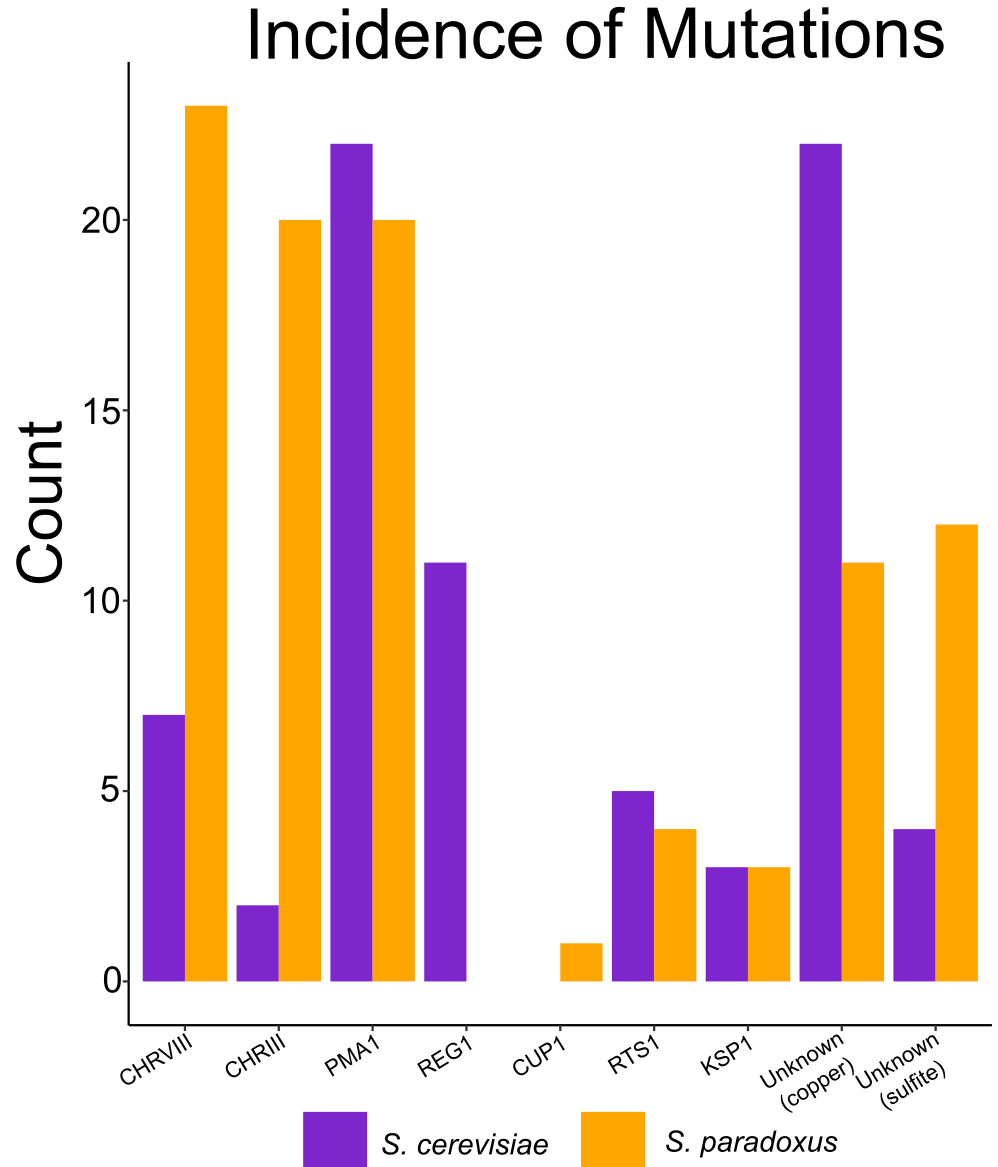


**Figure S3**. Incidence of the different mutant classes assigned as causal among sequenced mutants. Note that although there are two instances of chromosome III aneuploidy among *S. cerevisiae mutants*, this mutant class did not meet the significance threshold for causal aneuploidies in *S. cerevisiae* (p = 0.36).


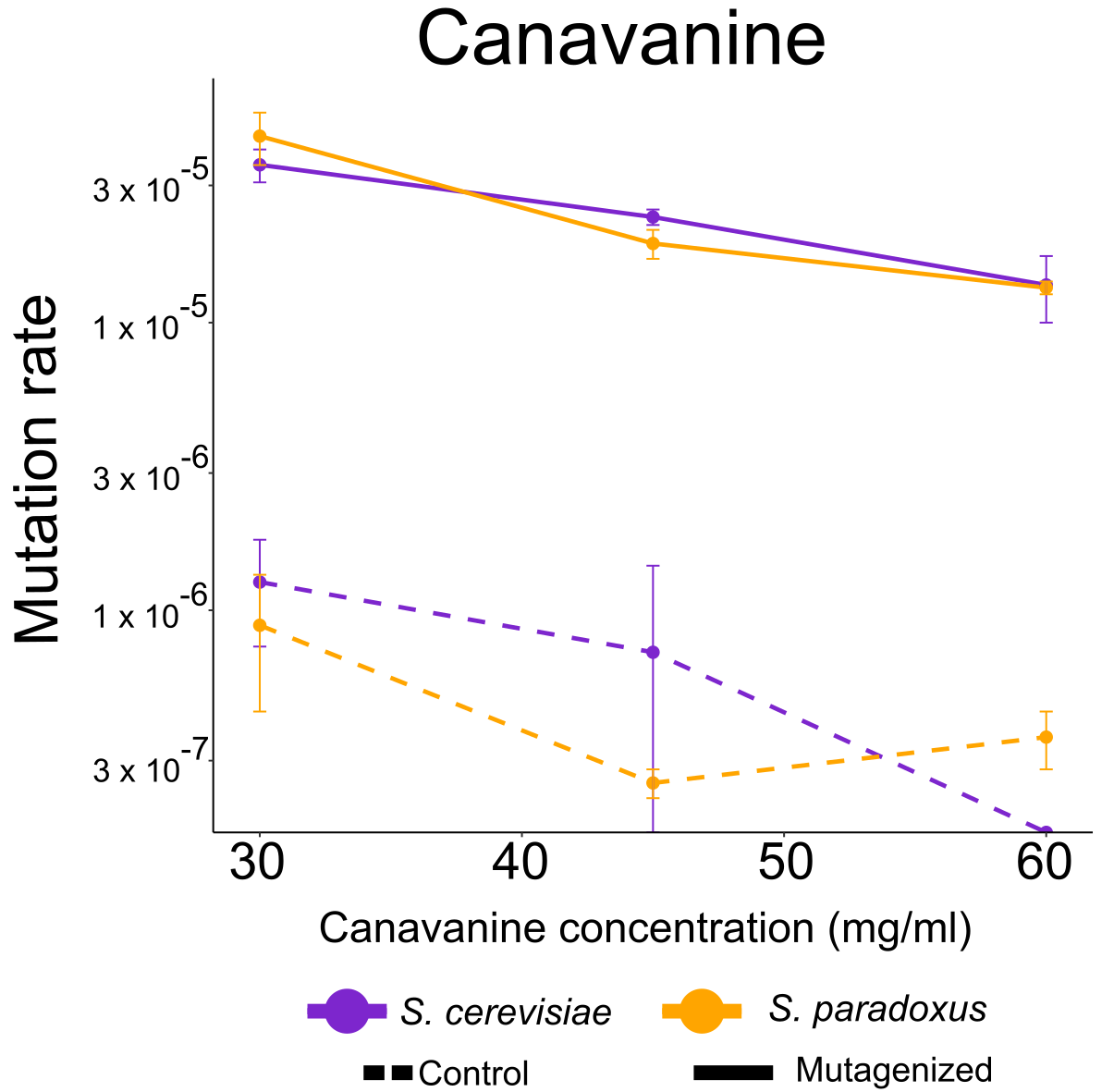


**Figure S4.** Canavanine mutation rates. Mutant recovery rates on three different concentrations of canavanine measured as colonies recovered divided by the number of cells plated. Mutagenized pools are denoted by solid lines and controls (mock mutagenized) are denoted by dotted lines. Error bars represent the standard deviation of the measurements of the different ancestral strains.


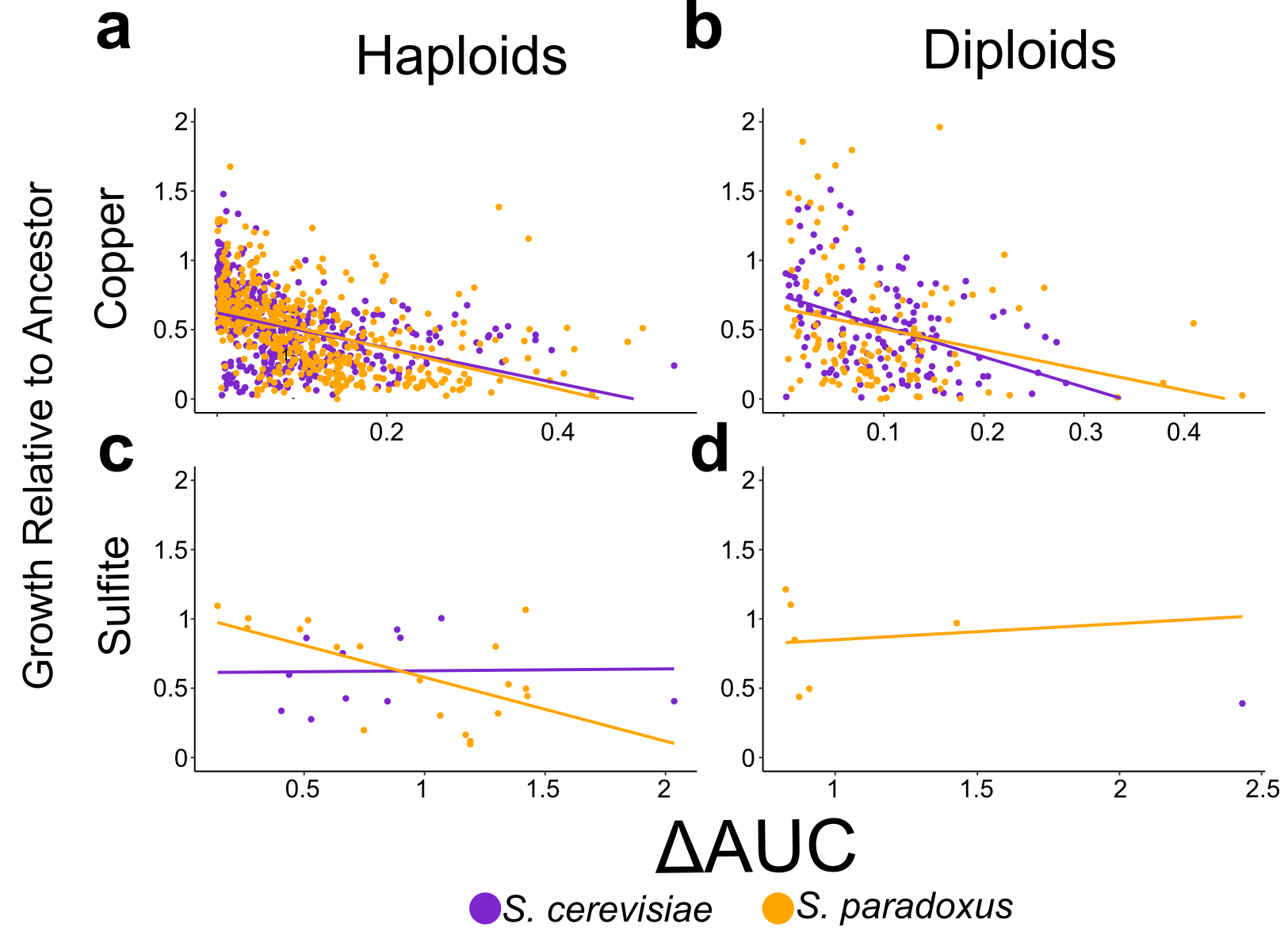


**Figure S5**. Average costs relative to the ancestor in permissive conditions in relation to mutational effect size. Panels show copper mutants (a, b) and sulfite mutants (c, d) for haploids (a, c) and diploids (b, d) of *S. cerevisiae* and *S. paradoxus.* Lines represent linear regressions for each species.


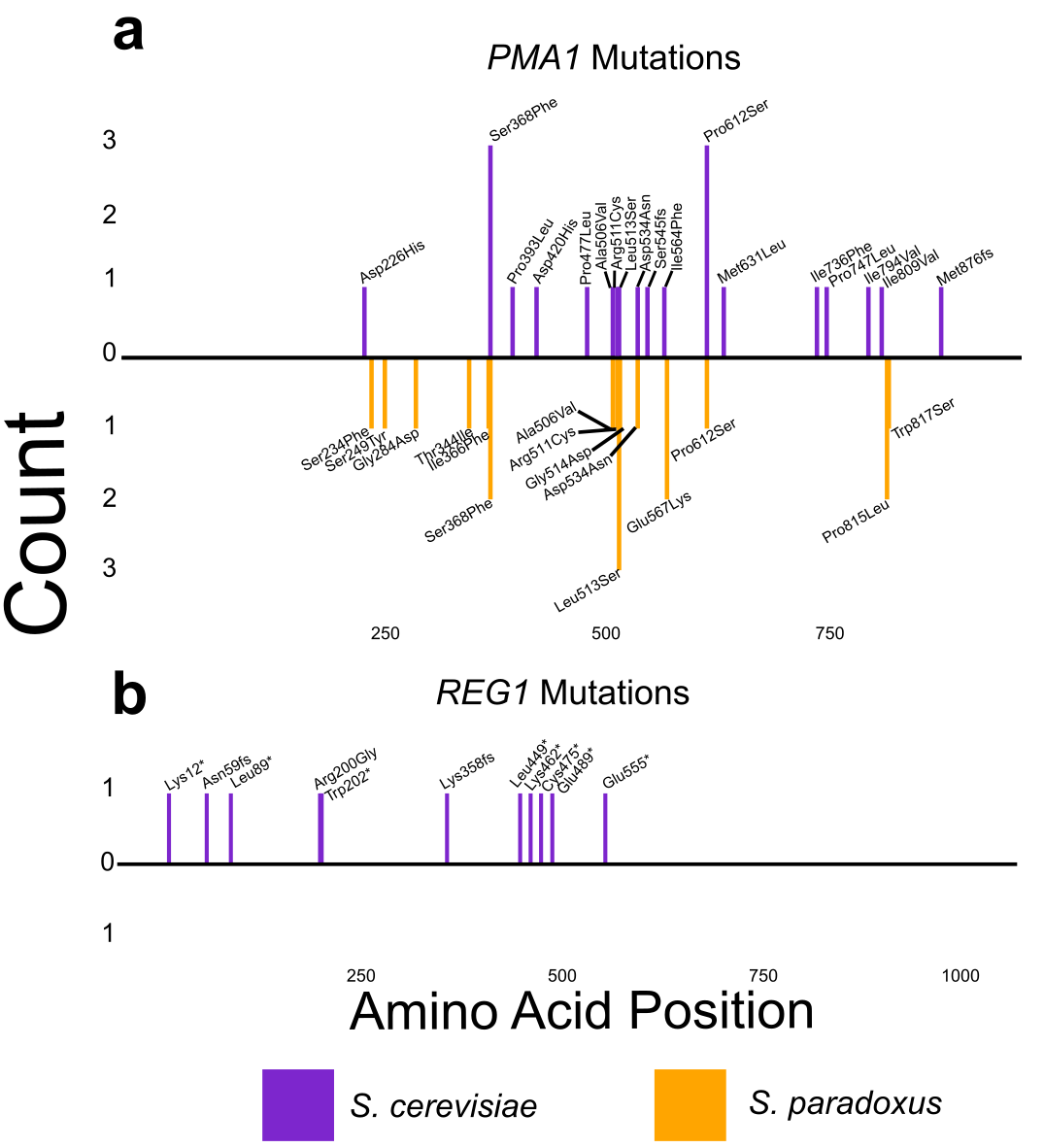


**Figure S6**. Location, identity, and frequency of (a) *PMA1* and (b) *REG1* mutations recovered among strains sequenced in this study. Nonsense mutations are indicated by “*”.


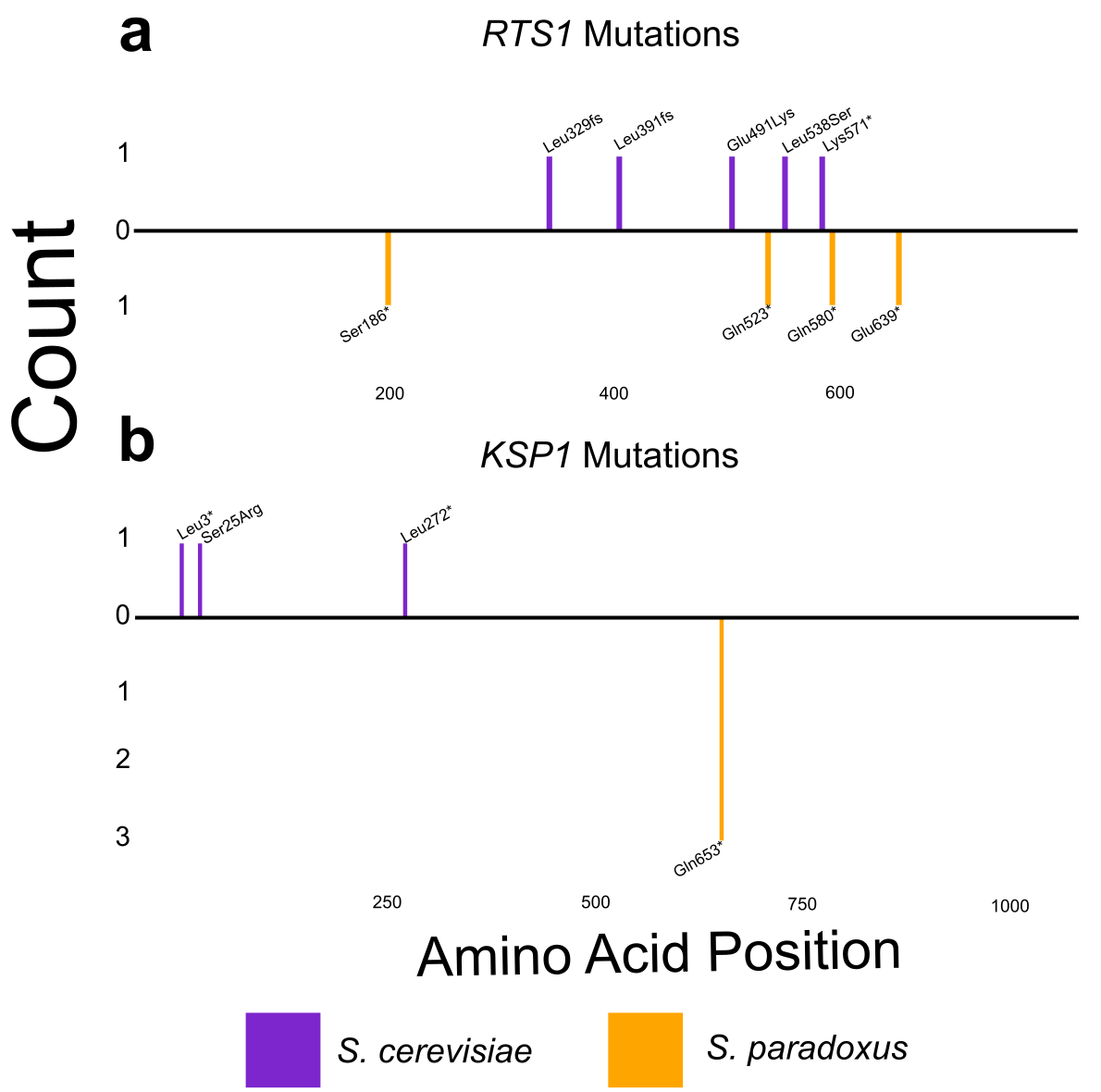


**Figure S7**. Location, identity, and frequency of (a) *RTS1* and (b) *KSP1*, mutations recovered among strains sequenced in this study. Nonsense mutations are indicated by “*”. Note that although three sequenced isolates of *S. paradoxus* had identical changes in *KSP1*, these are not presumed to have arisen independently (Table S3).
